# Evidence for Varied Aetiologies Regulating the Transmission of Prion Disease: Implications for Understanding the Heritable Basis of Prion Incubation Times

**DOI:** 10.1371/journal.pone.0014186

**Published:** 2010-12-02

**Authors:** Conrad O. Iyegbe, Oduola O. Abiola, Chris Towlson, John F. Powell, Steven A. Whatley

**Affiliations:** 1 Psychosis Centre, Institute of Psychiatry, King's College London, London, United Kingdom; 2 Department of Neuroscience, Institute of Psychiatry, King's College London, London, United Kingdom; 3 Experimental Neurochemical Pathology Laboratory, Institute of Medicine, Universiti Brunei Darussalam, Negara Brunei Darussalam; Mental Health Research Institute of Victoria, Australia

## Abstract

**Background:**

Transmissible Spongiform Encephalopathies (TSEs) are a group of progressive fatal neurodegenerative disorders, triggered by abnormal folding of the endogenous prion protein molecule. The encoding gene is a major biological factor influencing the length of the asymptomatic period after infection. It remains unclear the extent to which the variation between quantitative trait loci (QTLs) reported in mouse models is due to methodological differences between approaches or genuine differences between traits. With this in mind, our approach to identifying genetic factors has sought to extend the linkage mapping approach traditionally applied, to a series of additional traits, while minimising methodological variability between them. Our approach allows estimations of heritability to be derived, as well as predictions to be made about possible existence of genetic overlap between the various traits.

**Methodology/Principal Findings:**

Our data indicate a surprising degree of heritability (up to 60%). Correlations between traits are also identified. A series of QTLs on chromosomes 1, 2, 3, 4, 6, 11 and 18 accompany our heritability estimates. However, only a locus on chromosome 11 has a general effect across all 4 models explored.

**Conclusions/Significance:**

We have achieved some success in detecting novel and pre-existing QTLs associated with incubation time. However, aside from the general effects described, the model-specific nature of the broader host genetic architecture has also been brought into clearer focus. This suggests that genetic overlap can only partially account for the general heritability of incubation time when factors, such as the nature of the TSE agent and the route of administration are considered. This point is highly relevant to vCJD (a potential threat to public health) where the route of primary importance is oral, while the QTLs being sought derive exclusively from studies of the ic route. Our results highlight the limitations of a single-model approach to QTL-mapping of TSEs.

## Introduction

The *Prnp* locus has long been established as the major genetic determinant of Transmissible Spongiform Encephalopathy (TSE) incubation time, however its effects do not fully explain the variance associated with this trait, thus other critical factors must also play an important role in the aetiology of these disorders [1], [2]. TSE pathogenesis has been shown to be heavily dependent on intrinsic factors specific to the strain of the agent, the route of transmission [3] and dose of infection [4]. Since the identification of the prion protein gene, more recent studies have established that the risk from acquired prions is mitigated by host genes other than *Prnp* [2], [5]. Although incubation period phenotypes in the TSEs are the product of multiple small gene effects, their overall impact may be cumulatively substantial. Exactly how substantial (ie. their collective heritability) is a question to date unexplored for acquired TSEs on account of their rarity and the challenges posed by their potentially long incubation periods. Prion research has benefitted from the success of linkage studies conducted in the mouse. These have highlighted several regions associated with the incubation period phenotype [5]. Additionally a recent genome wide association study of human TSEs cases conducted by Mead et al [6] identified common variants for age of onset and death, two corollaries of the incubation period phenotype.

The contrasting genetic approaches of linkage and association are complementary and normally explore competing hypotheses regarding the underlying genetic architecture of disease progression. They provide a means by which to interrogate the genome for both common variants of small effect and rarer variants of intermediate/large effects. Current evidence suggests that the two models are not mutually exclusive and may coalesce [6], [7], [8].

Methodological variability precludes most meaningful comparisons between previous linkage efforts, as studies differ by either the chosen genetic background, prion source, or the host adaptation status of the agent [5]. However the frequency with which QTLs have been successfully identified across these studies advances the case for a genetic basis independent from *Prnp* while, the variability between the loci reported gives some indication of the scale on which influential variants may exist. However still this leaves little objective rationale for distinguishing the more empirically relevant loci from those whose effects are confined to only certain genetic backgrounds.

In all, 5 previous linkage studies have implicated QTLs on 12 different chromosomes [5], [7], [8], [9], [10]. The more recurrent among these loci such as chromosome 11, provide a stronger rationale for targeted fine mapping approaches. These previous studies are all based on intracranial (*ic*) transmission, which involves the introduction of prion material directly to the brain. This paradigm may model infection arising from exposure to larger peripheral doses of infectivity, as the relatively long period of replication in the spleen that would normally be seen in peripheral administration is bypassed in this instance, suggestive of direct neuroinvasion [11]. Peripheral neuroinvasive routes are specifically relevant to the transmission of human acquired TSE forms such variant Creutzfeldt-Jakob Disease (vCJD) [12], which poses a potentially major risk to public health. The question of whether *ip* transmissions have a unique genetic basis or one that may be shared across routes and between agents has to date not been addressed. A comparative approach to defining genetic commonality between traits may therefore be useful for classifying QTLs according to their empirical relevance to TSE transmissions.

The BXD set of recombinant inbred mice were the genetic cross chosen for this study. TSE incubation QTLs have not been explored in this particular background, therefore it is of interest to determine whether QTLs identified in previous studies could also be detected here. The BXDs derive from an F2 intercross of C57BL/6 and DBA/2J progenitors [13]. By definition, recombinant inbred lines are genetically homozygous at each locus and genotypes for nearly 4000 loci are readily-available. In this case both progenitor strains are homozygous for the same short-incubating (*s7*) prion allele. This effectively eliminates genetic variation at this locus from the study. The recombinant inbred (RI) method exploits the fact that the individuals within each line are genetically identical, hence trait values are based on group means as opposed to individual measurements. This makes QTL detection feasible even for traits with low heritability, due to a compensatory gain in the accuracy of phenotype measurement.

Additionally, the integrative ability of the RI approach can be exploited in order to produce genetic correlations between traits which allow both estimations of genetic overlap and heritability of traits. With this in mind the goals of the work were as follows:

- To determine the total contribution made by genetic variants of small effect (QTLs) through estimations of trait heritability.
- To estimate possible overlap between the genetic architectures regulating different transmissions by genetic correlation.
- To identify new QTLs for different treatment conditions, including the transmission of natural BSE, the *de facto* experimental model for vCJD.
- To provide validation of any previously identified QTL candidates.

## Results

### The distribution of BXD incubation periods

**Figure 1:**
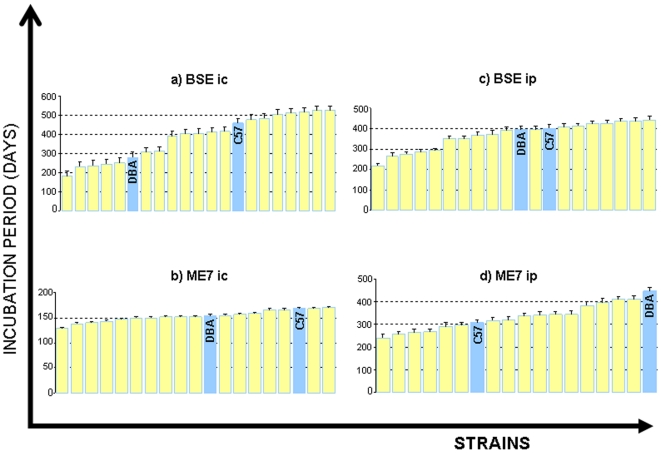
Means and s.e.m of strain incubation periods are given for the four agent/route combinations shown in **table 1**. Bovine Spongiform Encephalopathy (BSE), intracranial administration (ic), intraperitoneal administration (ip).

Raw phenotype data for the BXD lines and the progenitor strains C57BL/6 and DBA/2J are presented in table 1. An illustrative representation is shown in Figure 1. Plots of BXD strain means in Fig. 1 are continuous in nature, confirming the polygenic nature of TSE incubation times in the BXD genetic background. In all cases strain means extend outside the range of the progenitors. Relative differences between strain mean rank for each trait (table1) allude to trait-dependent genetic involvement.

**Table 1:** BXD strain means for prion disease incubation time.

Strain	BSE *ic* *n* = 19	BSE *ip* *n* = 18	Me7 *ic* *n* = 17	Me7 *ip* *n* = 16
C57BL/6J	460±49 (*n* = 9)	404±39 (*n* = 15)	167±13 (*n* = 13)	307±26 (*n* = 17)
DBA/2J	282±13 (*n* = 18)	397±51 (*n* = 15)	153±11 (*n* = 16)	448±110 (*n* = 12)
BXD1	312±92 (*n* = 9)	-	-	-
BXD2	411±48 (*n* = 14)	374±51 (*n* = 15)		345±46 (*n* = 10)
BXD6	523±41 (*n* = 13)	348±76 (*n* = 10)	152±14 (*n* = 10)	336±18 (*n* = 14)
BXD8	405±15 (*n* = 12)	369±17 (*n* = 11)	142±4 (*n* = 8)	267±31 (*n* = 12)
BXD9	513±52 (*n* = 12)	438±31 (*n* = 17)	165±8 (*n* = 12)	411±26 (*n* = 16)
BXD11	504±51 (*n* = 16)	443±30 (*n* = 15)	154±7 (*n* = 19)	398±65 (*n* = 14)
BXD12	185±10 (*n* = 10)	265±57 (*n* = 14)	152±17 (*n* = 15)	265±33 (*n* = 12)
BXD14	478±40 (*n* = 10)	412±33 (*n* = 14)	150±7 (*n* = 10)	291±47 (*n* = 8)
BXD18	238±10 (*n* = 5)	217±16 (*n* = 5)	138±16 (*n* = 5)	-
BXD19	483±43 (*n* = 17)	423±32 (*n* = 18)	170±9 (*n* = 14)	343±44 (*n* = 19)
BXD21	232±45 (*n* = 14)	391±24 (*n* = 12)	159±5 (*n* = 15)	341±16 (*n* = 12)
BXD22	417±79 (*n* = 12)	399±4 (*n* = 12)	167±14 (*n* = 12)	317±24 (*n* = 16)
BXD23	308±79 (*n* = 16)	349±74 (*n* = 12)	141±13 (*n* = 13)	241±51 (*n* = 18)
BXD24	517±21 (*n* = 9)	410±25 (*n* = 16)	152±21 (*n* = 16)	319±51 (*n* = 15)
BXD25	245±70 (*n* = 11)	291±78 (*n* = 8)	149±11 (*n* = 10)	296±27 (*n* = 6)
BXD27	523±18 (*n* = 14)	428±44 (*n* = 17)	166±10 (*n* = 11)	410±34 (*n* = 15)
BXD28	251±107 (*n* = 10)	273±62 (*n* = 8)	129±6 (*n* = 9)	-
BXD31	406±21 (*n* = 14)	286±47 (*n* = 12)	157±14 (*n* = 10)	256±23 (*n* = 14)
BXD32	391±189 (*n* = 9)	434±23 (*n* = 17)	148±11 (*n* = 14)	385±110 (*n* = 8)
Expt Mean	385±53	368±38	153±11	332±43

Incubation period data are given as mean ± s.d. for each trait. Bovine Spongiform Encephalopathy (BSE), intracranial administration (ic), intraperitoneal administration (ip).

The lowest overall variances are seen in Me7 *ic*, (fig. 1b) (129 to 170 days) reflecting the host-adapted nature of this agent. C57 appears to be the less susceptible of the two progenitors overall, as incubation periods for this strain generally exceed those of DBAs, (the only exception to this is Me7 *ip*). Internal reliability was assessed from BXD incubation period data using the Split-Half method [13]. A split-half coefficient of 0.7 and above equates to an s.e.m. of over half (0.55) a standard deviation. The mean Split-Half coefficient for all traits is 0.90 (all traits). The average reliability for any single trait is over 0.86 or greater (Table S1).

### Heritability Estimations

Heritability in RI mice is determined as one half of the variance between strain means. Genetic effects in BXDs are predominantly additive in nature, which permits the calculation of narrow-sense heritability; ie, the proportion of phenotypic variance ascribable to additive gene effects (see methods).

The heritabilities shown in table 2 are modest, but provide evidence that a large component of TSE incubation period may be influenced by a genetic architecture independent from *Prnp*. Heritabilities range from 0.30 (Me7 *ic*) to 0.60 for Bovine Spongiform Encephalopathy given *ic* (BSE *ic*). This suggests between 30 to 60% of phenotypic variance may be due to variation in genes. The upper limit of 0.6 is somewhat surprising given that direct effects from *Prnp* (the major genetic locus for the disease) have been excluded by experimental design. The extent to which variance between mouse strains (on which these estimates are based), may be impacted on by factors pertaining to litter or maternal rearing differences is beyond the scope of this study. Such interference may occur by way of various epigenetic processes. These may include the chemical modification of DNA (genomic imprinting) or interference of messenger RNA. The reader should refer to [14] for an outline of the theory and [8] for a direct account of the impact of maternal factors on TSE incubation time, in an experimental setting. Similar phenomena in humans may also help to explain discordances in age at onset of up to 8 years in twins carrying the inherited prion mutation P102L [15], [16]. In this study such effects are assumed to have no net effect on phenotype.

**Table 2:** Derived estimates of TSE heritability for the BXDs.

Parameter	BSE *ic*	BSE *ip*	Me7 *ic*	Me7 *ip*
Mean Variance_WithinStrain_	4697	2068	142	2125
Variance_Between Strain_	13371	4840	123	3033
Heritability_Narrow sense_	0.59	0.54	0.30	0.42

Trait variances and heritability estimations (narrow-sense). Heritabilities were calculated from incubation time data, having first established the independence of variance from group size.

Broadly speaking, the range of heritabilities shown in table 2 suggests that for some traits the cumulative effect of QTLs on incubation time may *exceed* the major locus effect of *Prnp*. However neither the existence nor involvement of trans-acting regulators of *Prnp*, (which could potentially bias these heritability estimates) can be ruled out based on our data.

### Genetic correlations

Genetic correlations were performed in order to investigate the possible involvement of QTLs across multiple transmission models.

The correlations in table 3 estimate the total proportion of host genetic effects that may be shared by different models. The data suggest a complex picture in which traits are underpinned by both shared and unique genetic effects.

**Table 3:** Inter-Trait genetic correlations.

	BSE ip	Me7ic	Me7ip
**BSE ic**	0.70**	0.52*	0.29
**BSE ip**		0.60*	0.67*
**Me7ic**			0.38

Genetic correlations were calculated using BXD incubation data for TSE traits.

*p = 0.05,

**p = 0.001.

Some comparisons reach statistical significance. For example, the correlation between traits exclusively involving either the *ip* or the *ic* route reaches 0.52, p<0.05 (Me7 ic versus BSE ic) and peaks at 0.67, p<0.05 (for BSE *ip* versus Me7 *ip*).

The latter correlation in particular suggests that a partially conserved genetic infrastructure regulates transmission through the ip route and seemingly provides evidence of a dynamic effect across a range of agents, which encompass differences in host-adaptation.

A strong correlation between BSE *ic* and BSE *ip* (r = 0.702, p = 0.001) contrasts with the low level of correlation found for Me7 ic and Me7 ip (r = 0.38). The case for genuine disparity between the levels of genetic conservation across routes for natural and host-adapted isolates would benefit from further replication of these findings. Curiously, a greater degree of overlap (r = 0.6) *is* observed between Me7 *ic*, and BSE *ip* (p<0.05) than exists between the two traits involving Me7. This is notable as it suggests that tight conservation of the supporting architecture can potentially transcend seemingly random differences in route and agent. However it is also surprising, given that the relatively high correlation is found to be unique when the reciprocal comparison (BSE ic vs Me7 ip) is made (r = 0.29).

Unfortunately the study design employed is not well-suited for discerning what (if anything) is so special about the combination of Me7 *ic* and BSE *ip*. Moreover, due to the absence of a cloned BSE isolate (such as 301C) from this study, we have been unable to discern whether the BSE correlation effect is a *pre* or *post* species barrier phenomenon.

Despite the high level of statistical significance achieved the level of genetic overlap estimated for BSE traits is far from perfect. The large number of QTLs likely to be operating on this complex trait dictates that even smaller deviations from a perfect correlation could be significant from a biological perspective.

### Mapping of BXD incubation periods

#### Summary

Loci meeting nominal significance criteria were interrogated for superimposed effects from other background loci (composite interval mapping). Results for the composite interval mapping (CIM) and simple interval mapping (IM) are given in table 4 for markers achieving suggestive-level linkage (Table 4).

**Table 4:** Interval mapping results for the BXDs.

QTL Location	Condition	Marker	Position (cM)	Probability (LRS)		Additive Effect (days)		Direction	
				IM	CIM	IM	CIM	IM	CIM
Chromosome 4	BSE ic	D4Mit204	61.9	16.9*	36.4***	86.5	59.3	C57	C57
Chromosome 4	Me7 ic	D4Mit204	61.9	11.4*	28.2***	7.5	5.7	C57	DBA
Chromosome 6	BSE ip	D6Mit29	36.5	15.3*	24.7**	51.1	51.5	DBA	DBA
Chromosome 6	Me7 ic	D6Mit67	41.5	13.2*	41.1***	8.0	12.8	DBA	DBA
Chromosome 6	Me7 ip	D6Mit29	36.5	8.1*	46.2***	39.2	45.7	DBA	DBA
Chromosome 10	BSE ic	S10Gnf020.445	14.8	18.1*	46.1***	97.9	78.2	C57	C57
Chromosome 11	BSE ip	D11Mit179	52.0	12.8*	23.8**	49.5	32.4	DBA	DBA
Chromosome 18	Me7 ip	D18Mit19	4.0	15.2*	43.8***	44.7	52.3	DBA	DBA

Simple and composite mapping data for TSE incubation times. Only the peak marker within each QTL region is shown in each case. Asterisks denote genome-wide significance, thresholds for which were calculated by 10,000 permutations of trait data:

*Suggestive,

**Significant;

***Highly significant thresholds correspond to the 37^th^, 95^th^ and 99.9^th^ percentiles, respectively. Allele direction indicates the allele mediating the increase in incubation time.

The number of QTLs found exceeds theoretical expectation as, only one false positive is anticipated per genome-wide scan at the suggestive level [17]. Among the composite mapping results are three examples where QTLs appear to have effects across traits.

BSE *ic*; Me7 *ic* (chromosome 4)

BSE *ip*; Me7 *ic* (chromosome 6)

Me7 *ip*; BSE *ip* (chromosome 6)

Though the possibility of genetic heterogeneity within QTLs is not excluded, the data suggest correspondence between these results and the empirical scheme of genetic overlap outlined by table 3. Table 4 gives no indication of any delineation between natural and host-adapted TSE strains. Loci such as D4Mit204 and D6Mit29 are common to both.

The refining effect of CIM, when applied to simple IM data is also demonstrated in Table 4. Adjustments to LRS score, additive affect and allelic direction are all noted (all linkage statistics were improved by the CIM models applied). These refinements are non-formulaic and depend on the composition of the background construct, hence the largest LRS score of 46.2 (D6Mit29, Me7 *ip*) is actually derived from the lowest IM score shown in this table (ie. 8.1). All loci remained significant after Bonferroni multiple testing correction was applied to CIM scores.

### F2 Analysis

#### Qualitative comparisons of F2 and BXD phenotype data

The strongest QTL candidates from the initial BXD mapping data were selected for confirmatory follow-up in an F2 population. The F2 sample were derived from an identical genetic background to that of the BXDs. Transmissions of TSE material were carried out using the same combinations of agent and route described for BXDs (see methods). Incubation data was normalised where required (see methods).

**Figure 2:**
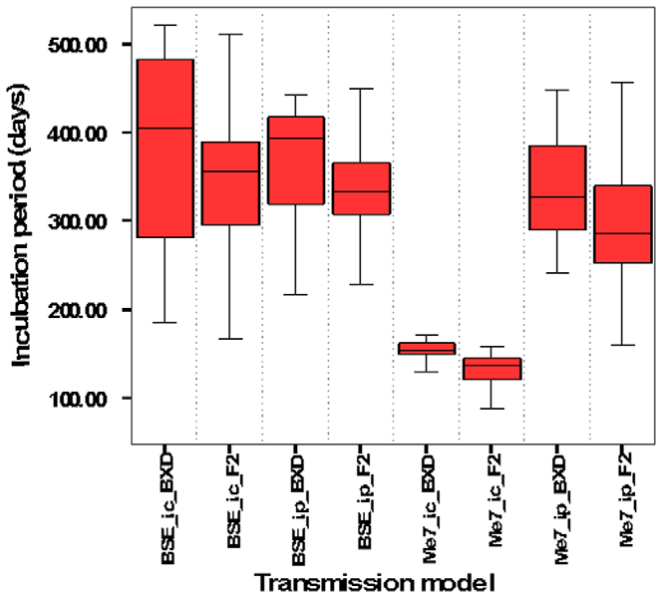
Boxplot comparison of BXD strain and F2 incubation period data by experiment. Black horizontal lines within the coloured bars give the median in each experiment. Lower and upper boundaries of blue bar represent 25^th^ and 75^th^ percentiles, respectively. Lower and upper tails demarcate largest and smallest values within 95% confidence interval. Me7 *ic* and Me7 *ip* transmissions produce significantly different incubation times between BXDs and F2s.

Data in Table 5 and the graph in fig. 2 highlight the overall similarity between BXD and F2 incubation periods. To help qualify this statement, the calculated Pearson's *r* between BXD and F2 trait means is 0.813 (P = 0.049). Additionally, trait means follow identical rank order to those obtained in BXDs.

**Table 5:** Comparison of means for BXD and F2 incubation periods.

Experiment	Mean BXD Incubation period (Days±s.e.m)	Mean F2 Incubation period (Days±s.e.m)
BSE ic	386±26.5	345±3.5
BSE ip	364±16.4	337±3.3
Me7 ic*	152±2.7	129±2
Me7 ip*	326±13.8	292±6

Phenotypic comparisons of BXD and F2 means. This data is based on data presented in table 1, although in this instance the progenitor strains are omitted from calculated values to ensure an unbiased comparison of mean phenotype values. Data for the progenitor strains.

*BXD and F2 means differ significantly at the P<0.01 level (T-test).

The level of correlation found suggests that for TSE incubation periods, the comparatively lower phenotyping accuracy of F2s can be overcome with a sufficiently large F2 cohort. Such a strategy may be attractive for avoiding problems associated with breeding and maintaining a large RI mouse colony.

Despite the overall similarity between BXD and F2 mouse panels, individual discrepancies are noted: The panels differ for Me7 *ic* and Me7 *ip* transmissions at the P<0.01 level. BXD traits values are derived from strain means thus, given the high reliability scores associated with these values (data not shown), it is more likely that individual discrepancies are the result of greater phenotyping error among F2s.

#### Results of F2 interval mapping

QTLs reaching minimum suggestive (p = 0.63) significance (composite model) are presented. In all, nine QTLs reach our significance criteria, (table 6). As in the BXD analysis, the yield of suggestive QTLs is above the expected false positive rate (one per genomewide scan). Me7 *ic* is the only trait for which QTLs are not detected, this reflects the low phenotypic variance observed in F2s. In the case of F2 interval mapping the effects of composite mapping on linkage peaks is much less dramatic than is observed in the BXDs. CIM should ideally be performed after ensuring that marker coverage is sufficient to provide an unbiased estimation of background effects. However this study has only tested those markers chosen to flank BXD-mapped QTLs.

**Table 6:** QTL mapping data for F2s.

Location (Chr)	Transmission	Mapping model	F2 LRS Statistics	95% Support Interval (1-LOD)	F2 Additive Effect (Standardised)	F2 Dominance effect (Standardised)
**1**	BSE ic	Simple	10.8*		0.24	−0.11
		Composite	12.9*	37.8–61.1 cM	0.27	−0.15
	BSE ip	Simple	11.7*		0.15	0.71
		Composite	10.3*	>37.8 cM	0.28	0.38
	Me7 ip	Simple	13.1**		−0.44	0.46
		Composite	9.9*	21.7–38.1 cM	−0.43	0.37
**2**	BSE ic	Simple	5.6		−0.23	−0.16
		Composite	7.5*	1.0–25.3 cM	−0.27	−0.11
	BSE ip	Simple	10.0*		−0.31	0.28
		Composite	9.9*	20.0–54.0 cM	−0.29	0.32
**10**	Me7 ip	Simple	8.3*		−0.33	−0.34
		Composite	7.6*	17.0–66.0 cM	−0.47	−0.91
	BSE ip	Simple	7.9*		0.30	−0.70
		Composite	7.0*	>10.0 cM	0.30	−0.66
**4**	BSE ic	Simple	13.4*		−0.08	−0.39
		Composite	10.5*	65.6–68.7 cM	−0.07	−0.37
**6**	BSE ic	Simple	11.6*		0.06	0.52
		Composite	7.6*	46.3–51.9 cM	0.09	0.48

Summary of QTLs identified by composite mapping F2 incubation data.

* = Suggestive.

** = Significant genome-wide probability. Additive and dominance effect were calculated under an unconstrained genetic model and standardised by dividing by the standard deviation of the corresponding raw data. Minus values refer to the DBA direction, positive values refer to C57BL.

QTLs on chromosomes 1, 2 and 10 are linked with more than one trait. These loci demonstrate a corresponding level of allelic heterogeneity. This, along with the observation of genetic correlations (table 3), supports the notion that QTLs may have multiple effects across the different TSE phenotypes explored (ie QTL pleiotropy). Effects such as those found on chromosome 2 vary between additive and recessive modes. As these characteristics depend on the surrounding genetic architecture, they are expected to vary for different transmissions.

The prevailing BXD account of genetic overlap between *ic* and *ip* indicated in tables 3 is substantiated somewhat by QTL data in Table 4. Table 6 offers further supporting evidence from F2 data. QTLs on chromosomes 1 and 2 indicate overlapping influences on both BSE traits. The chromosome 10 locus has a broad effect on *ip* transmissions that extends to natural (BSE) and host-adapted (Me7) isolates. This is contrasted against QTLs on chromosomes 4 and 6, which show specific influences on BSE *ic*.

#### Comparison of BXD and F2 mapping data

Our primary criteria for confirming (ie. replication) is contingent on synchrony between BXD and F2 loci in terms of both statistical significance and allelic direction.

Table 7 indicates that confirmation was not possible for 6 out of 7 candidate regions. The exception was chromosome 2, where the effect found on BSE *ic was* replicated. Failures are mostly due to an inadequate significance level being attained.

**Table 7:** BXD and F2 mapping data.

Location of Interval	Trait	Interval mapping model	Peak BXD LRS	F2 LRS Statistics	Additive effect direction bxd (After CIM)	Additive effect direction F2 (After CIM)
Chromosome 2 (1.0–27.3 cM)	BSE ic	Simple	5.6	2.6	DBA/2J	DBA/2J
		Composite	7.5*	20.1*		
Chromosome 4 (53.6–81.7 cM)	BSE ic	Simple	16.9*	13.4*	C57BL/6	DBA/2J
		Composite	36.4***	10.5*		
Chromosome 4 (53.6–81.7 cM)	Me7 ic	Simple	11.4*	5.5	DBA/2J^†^	C57BL/6
		Composite	28.2***	2.4		
Chromosome 6 (15.3–45.5 cM)	Me7 ic	Simple	13.2*	2.6	DBA/2J	C57BL/6
		Composite	41.1***	3.5		
Chromosome 10 (2.0–29.0 cM)	BSE ic	Simple	18.1*	1.8	C57BL/6	C57BL/6
		Composite	46.1***	3.3		
Chromosome 11 (40–65.0 cM)	BSE ip	Simple	12.8*	5.4	DBA/2J	DBA/2J
		Composite	23.8**	5.8		
Chromosome 18 (4.0–17.0 cM)	Me7 ip	Simple	15.2**	4.5	DBA/2J	C57BL/6
		Composite	43.8***	2.8		

BXD and F2 QTL mapping data are shown. Loci linked to trait above the suggestive threshold (composite mapping) in BXDs are presented with the corresponding LRS score reached in F2s (Composite interval mapping).

*Suggestive level,

**Significant level,

***Highly significant.

Loci such as chromosome 4 make for difficult interpretation of the data, as the locus is clearly implicated by both BXD and F2 BSE *ic* models, but falls short of our criteria, due to allelic discrepancies. As discussed above, this too may be a symptom of including a small and biased pool of markers in the background-effects model. In order to circumvent the effect of such constraints to QTL detection, the empirical importance of QTLs effect across BXD and F2s was also explored by meta-analysis.

### Meta-Analysis

#### Random Effects meta-analysis of BXD and F2 data

Meta-analysis is used in the context of this study to estimate the overall influence on incubation period of candidate regions under different experimental treatments. Our methodology, an implementation of the Random Effects model [18] incorporates heterogeneity within and between BXD and F2 to derive empirical estimates of effect size, along with associated confidence intervals and *p* values. The random effects model places greater emphasis on the consistency of effect sizes between studies. Full meta-analysis results are shown in tables S2 and S3, where estimations of the mean effect size (Pearson's *r*) are provided, along with standard errors and significance. Data are initially combined between BXDs and F2s (table S2) before aggregating data of traits to derive empirical estimates (table S3). One-tailed significance thresholds are derived from recommended F2 thresholds (two-tailed) for 2 degrees of freedom [17]. Thresholds are *p* 8.0×10^−4^ (suggestive) and *p* 2.6×10^−5^ (significant).

#### Meta-Analysis results

The high frequency of loci reaching suggestive (p = 8.0×10^−4^) significance implies relatively low genetic heterogeneity between effects found for BXDs and F2s. This further corroborates the phenotypic similarity suggested by table 5; as consistency of effects is the main basis for linkage under the random effects model. Loci reaching standard significance thresholds are summarised in table 8.

**Table 8:** Summary of important meta-analysis findings.

QTL Locus	Trait	Effect size, r	Genome-wide Significance
Chromosome 1	Me7 ic	0.20±0.06	Suggestive
	BSE ip	0.28±0.07	Suggestive
Chromosome 2	Me7 ic	−0.22±0.06	Suggestive
Chromosome 4	Me7 ic	−0.30 ±0.06	**Significant**
	BSE ic	−0.19±0.05	Suggestive
Chromosome 6	BSE ic	0.15±0.04	Suggestive
Chromosome 11	Me7 ic	−0.19±0.06	Suggestive
	BSE ip	−0.23±0.05	**Significant**
	BSE ic	−0.15±0.04	Suggestive
Chromosome 18	BSE ip	−0.22±0.05	**Significant**

Loci of varying significance identified by Random Effects meta-analysis are given with associated effect sizes. Signs indicate directionality of the allelic effect; ‘−‘ corresponds to DBA and ‘+’ corresponds to C57 alleles.

The application of a random effects meta-analytical method to BXD/F2 transmission data has resulted in ten traits meeting minimum criteria for linkage. Of these, three reach genomewide significance (ie. with P<2.6×10^−5^). These are:

Me7 *ic* on chromosome 4: *r* 0.30±0.06, *p* 4.0×10^−8^

BSE *ip* on chromosome 11: *r* 0.23±0.05, *p* 1.0×10^−5^

BSE *ip* on chromosome 18: *r* 0.22±0.05, *p* 1.9×10^−5^

The strongest overall effect is found at chromosome 4 (*r* = 0.30). This locus also achieves the highest overall significance, at 10^−8^. The ten hits are distributed across six intervals and apply to three out of four traits analysed (table S2). Not represented among them is Me7 *ip.* Paradoxically, the largest number of hits (four) is found for Me7 *ic*, a trait from which the lowest phenotypic variations were derived. The possibility exists that statistical significances associated with the trait Me7 *ic* merely reflect this low variation across BXDs and F2s given the emphasis of random effects methods on effect homogeneity. However, we fail to find evidence that the group of uncombined effect sizes found for this trait differs significantly from other traits analysed, *P*_two-tailed_ = 0.927 (*T-*test).

#### The chromosome 11 QTL demonstrates general influence on TSE incubation periods

The rate of linkage at any level of significance diminishes as traits are combined (table S3), thus we find evidence of just one generally-acting QTL; the linkage region on chromosome 11. Tabulated data for this region are given in table 9. The chromosome 11 locus is significant overall (P = 9×10^−10^). Additional significant effects are observed for ic and Me7 experiments (P = 2×10^−7^ and 7×10^−7^ respectively). Effects for BSE reach suggestive (P = 1×10^−4^). ip transmissions show only a trend towards significance (P = 0.002). This is due to the larger standard errors associated with these estimated effects. All are consistent in direction, with reductions in disease progression (which corresponds with lengthening incubation time) mediated by DBA alleles.

**Table 9:** Breakdown of meta-analysis effects found in chromosome 11 QTL region.

Agent	Combined r± s.e.m	*r^2^*	Direction	*P* Value
BSE	−0.19±0.05	0.04	DBA2/J	1×10^−4^ *
Me7	−0.18±0.04	0.03	DBA2/J	7×10^−7^ **
ip route	−0.18±0.06	0.03	DBA2/J	2×10^−3^
ic route	−0.18±0.03	0.03	DBA2/J	2×10^−7^ **
All Expts	−0.18±0.03	0.03	DBA2/J	9×10^−10^ **

Summary of combined effects at Chromosome 11 locus. Varying s.e.m sizes underlie the large differences in significance.

*Suggestive;

**Significance.

The *r* ^2^ (table 9) gives estimations about the proportional additive variances from the locus. We estimate the overall contribution to phenotypic variance to be about 3%.

## Discussion

A long line of previous work has already established non *Prnp*-linked genetic modifiers as important determinants of incubation time [2], [5], [7], [8]. As it is customary that the initial scientific rationale for such work is provided by analyses of heritability, it is slightly ironic that the estimation of heritability for human prion phenotypes has only very recently been possible [19]. One factor precluding heritability analyses of vCJD, a future potential public health risk, is its low recurrence rate among nuclear families; there has been just one case of vCJD recurring in a family (Source: European Centre for Disease Prevention).

The heritability of age at onset and death for the human *inherited* TSE form was recently determined to be 0.55 (95% CI 0.35–0.75) [19]. This value is comparable to our own estimates which derive from a related phenotype and are the first to be reported for an *acquired* TSE (*h^2^_narrow sense_* range  = 0.3 to 0.6). As in [19], our estimates exclude direct effects from *Prnp* and suggest a cumulatively modest contribution of non-*Prnp* effects. We demonstrate that they may nevertheless outweigh those of the only known major locus, in response to varying the parameters explored by this study.

Our QTL data, only partially justifies our findings in relation to heritability, thus we acknowledge and draw further attention to the many possible sources of bias on which such estimates may rely [14], [8]. Additionally further biases may result due to the relatively small proportion of the currently available BXD strains sampled in the study, irrespective of any phenotypic similarity observed between BXDs and F2s across traits (Pearson's *r* 0.813, p = 0.049). In this instance, the use of the expanded BXD set now available was not possible, but may well have resolved such curiosities as a recurrent but unconfirmed locus on chromosome 10 for BSE *ic* (BXD), Me7 *ip* (F2), and BSE *ip* (F2). Such patterns may not occur randomly and may actually reflect additional complexity at these loci. Evidence that the distribution of BXD and F2 QTLs in our data is non-random derives from the fact that:

- A combination of stringently employed mapping and meta-analytical methodologies have yielded many pre-existing QTLs known to be involved in controlling prion incubation times.
- There are a high number of F2 QTLs in relation to the much smaller hypothesis region tested (9 F2 QTLs were identified within the 27 marker loci tested, whereas 12 BXD QTLs were identified across 3795 tested marker loci). The F2 number is significantly higher than *a priori* expectations based on extrapolated BXD data (P*_Yates corrected χ^2^_*<<0.0001) and argues that the methodological procedures in the BXD analysis offer sufficient protection against Type 1 error in this study. This implies enrichment for genuine QTLs in BXD and F2 data as well as further complexity in the mechanism underlying these QTLs. That previously reported QTLs were detected by this study goes some way to reaffirming the validity of a novel suggestive (p<8×10^−4^) locus on chromosome 1 (35–62 cM) and a significant (p<2.6×10^−5^) locus on chromosome 18 (4–17 cM).

F2 mapping was, by itself, able to reproduce a locus on chromosome 2 specific to BSE *ic* that reached the genomewide threshold for suggestive significance (p = 0.63). That this locus did not survive the subsequent *random effects* meta-analysis procedure merely emphasises the heterogeneity between the BXD and F2 estimates and does not contradict the initial evidence suggesting linkage at this region. The chromosome 2 QTL lies approximately 50 centiMorgans (cM) upstream of *Prnp* (located at 75 cM). Independence of the chromosome 2 locus from *Prnp is* assumed. Among the loci identified for *ic* transmissions are a number of familiar linkage regions, (corresponding to regions of chromosomes 2, 4, and 11) previously linked to this route [5], [7].

We are the first study to attempt to quantify the genetic basis of aetiological overlap governing different routes and agents. Table 3 highlights the potential for strong overlap effects, even when traits differ simultaneously by host adaptation, route and agent. The caveat is that strong effects of route and agent on genetic influence, suggest that QTLs identified in one study cannot necessarily be extrapolated to transmission models based on different combinations of agent and route. Thus, while it may be too tempting to resist extrapolating previously reported data to the oral route (a route more relevant to the transmission vCJD) our data suggest that doing so may blur the little that we understand of the genetic processes regulating the transmission of vCJD and the potential risk to public health that they may represent. This is because even the strongest correlating traits in our study do not share more than 70% of genetic influences in common, while our lowest estimates suggest that the dissimilarity between transmissions can mean just 50% overlap or less, even between transmissions using a common administration route (table 3).

The overall implication is that any lingering uncertainty about the identity of risk variants in vCJD may be best tackled using mapping strategies that specifically target the more relevant oral route. Reassuringly, table 3 suggests some portion of genetic influence may be common to all traits. The emphatic nature of the meta-analysis result and other prior linkage evidence makes the chromosome 11 locus one prime candidate for mediating such a role. Meta-analysis also highlights a number of other significant candidate regions, which are summarised in table 8. Trait-specific QTL effects of varying significance were found on chromosomes 1, 2, 4, 6 and 18, after the merging of BXD and F2 data.

**Figure 3:**
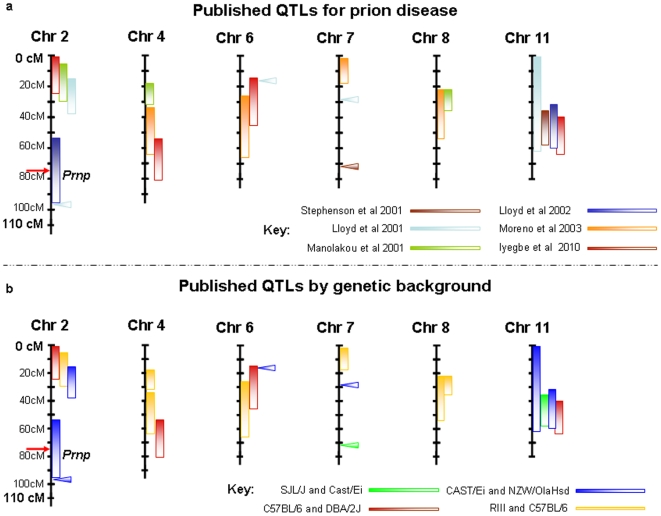
Summary of QTL intervals affecting TSE transmission by study (3a) and by genetic background (3b). Only chromosomes with multiple QTL assignments are presented. All intervals with the exception of Lloyd et al are calculated in the absence of background modifiers. Manolakou intervals are estimated using XYExtract V2.5 (2004) of Cleiton and Silva.

Our data represent the third independent report of a distinct TSE locus on chromosome 2 (fig. 3a). The linkage region overlaps with previous QTLs identified in [8] and [7]. Other QTLs located at the distal end of chromosome 2 have been identified by Lloyd et al [7], [9]. It is not clear to what extent these may be specific to the CAST/Ei x NZW/OlaHsd background in which they occurred. Interestingly one of the regions of Lloyd et al spans loci of both the prion protein (*Prnp*) and that of its paralogue Doppel (*Prnd*), although sequence symmetry between progenitor strains at this locus rules out effects from within either gene. We are the third group to independently report the existence of QTLs for prion disease on chromosome 11. Previous studies have already established this chromosome as a good linkage candidate. Crucially however, our meta-analysis data attaches empirical significance to this locus. This suggests wider involvement across a variety of other transmission types not characterised previously. Candidate-based explorations of the region have so far failed to reveal the origins of such effects [20].

Lloyd et al [9] (Table 10 and fig. 3a) observed similar genetic factors to those found in a previous investigation of the *ic* route [7] confirming the veracity of these loci. This result is perhaps not surprising given that each study uses the same genetic cross (NZW/Hsd and Cast/Ei) and only the TSE strain used (both host-adapted) varies. Moreno et al [10] and Manolakou et al [8] employ prion agents at different stages of host-adaptation. This may account for individual discrepancies between these results (in addition to methodological and environmental/epigenetic considerations) although, common regions on chromosomes 4 and 8 indicate a broader similarity between their findings.

**Table 10:** Summary of TSE linkage findings to date.

Study	Genetic Cross		Agent	Route	Interval	Maximum effect size (*r*)
	Susceptible	Resistant				
**Stephenson 2001**	**SJL/J**	**Cast/Ei**				
	105 Days	172 Days	Chandler Scrapie	ic	Chr10: 60 cM	-
					Chr11: 37–57 cM	0.41
					Chr7: 72.4 cM	-
					Chr9: 17–26 cM	-
					Chr18: 24 cM	-
					Chr19: 16–24 cM	-
**Lloyd et al 2001**	**NZW/OlaHsd**	**Cast/Ei**				
	108 Days	188 Days	Chandler Scrapie	ic	Chr2: 17–37 cM	0.27
					Chr11: 1–65 cM	0.50
					Chr12: 4–34 cM	0.26
					Chr6: 17.5 cM	0.13
					Chr7: 28.4 cM	0.14
**Manolakou et al 2001**	**RIII**	**C57BL**				
	442 Days	540 Days	BSE	ic	Chr2: 6.4–29 cM	0.23
					Chr8: 22–35 cM	0.23
					Chr4: 19.9–33.6 cM	0.23
					Chr15: 17.2–53.4 cM	0.23
**Lloyd et al 2002**	**NZW/OlaHsd**	**Cast/Ei**				
	133 Days	181 Days	3rd Passage BSE	ic	Chr2: 54–94 cM	0.53
					Chr11: 32–62 cM	0.39
**Moreno et al 2003**	**RIII**	**C57BL**				
	161 Days	167 Days	Me7	ic	Chr4: 34–64 cM	0.22
					Chr5: 51–72 cM	0.36
					Chr6: 25–67 cM	0.30
					Chr7: 2–18 cM	0.22
					Chr8: 22–64 cM	0.26
					Chr17: 7–21 cM	0.22
					Chr17: 36–46 cM	0.26
**Iyegbe et al 2009**	**DBA/2J**	**C57BL/6**				
	278 Days	454 Days	BSE	ic	Chr2:1–25.3 cM	0.11^a^
**Iyegbe et al 2009**	**DBA/2J**	**C57BL/6**				
	351 Days	319 Days	All	All	Chr11:40–65 cM	0.17^a^

Summary of all TSE QTLs identified to date. Effect sizes are calculated from percentage variances reported in published data. These are unconstrained estimates which exclude the effects of background markers.

a Derived from BXD and F2 data combined by meta-analysis (random effects) methods.

Previous studies show a bias towards C57-derived genetic backgrounds. Of the QTLs shown in fig. 3b we see that regions on chromosomes 2, 6 and 11 have the most general influence across varied genetic backgrounds. The chromosome 11 has already been mapped in two non C57-derived backgrounds, while loci on chromosomes 2 and 6 have been identified in the NZW/Hsd and Cast/Ei genetic cross.

A recent GWA study of human TSEs draws attention to novel common variants associated with clinical phenotypes, implicating genes such as *STMN2* and *RARB*. In addition, previously uncharacterised variants have also been discovered within *PRNP* [6]. The study identified two loci of genome-wide importance, one in strong LD with the major locus at codon 129 and another found in the intergenic region between *RARB* and *THRB*. The other genome-wide significant finding in this study highlighted a locus for orally acquired TSEs (vCJD and Kuru), using meta-analysis. Underlying genetic heterogeneity found between TSE categories in this study mirrors our own findings. We assume this to reflect that factors such as agent, dose and route also have an important role in aetiological determinism and its corresponding genetic framework.

The low recurrence rate of familial vCJD precludes studies of human linkage, in which rare disease loci may be detected by virtue of their increased occurrence among affected relatives. This and the low global incidence of vCJD, means increasingly-available next-generation sequencing methods cannot yet be fully exploited to define the possible contribution of rare variants to the TSEs. Recent progress made, across psychiatry in particular, has helped to demonstrate the pathogenic potential of this variant class [21]. Meanwhile, the continued application of linkage studies to murine proxies must continue in order to provide further cues for fine-mapping and positional cloning work. Gene targets identified by such approaches can advance human prion research by exploiting synteny between murine and human biological systems. QTL mapping in Heterogeneous Stock mice offers superior resolution to the RI approach and use of this genetic resource has the potential to expedite the search for QTLs by reducing the size of candidate regions to relatively few genes. Such approaches have been applied to great effect and have heralded the first wave of gene candidates and confirmed trait loci [22], [23] to derive from murine QTL mapping of the TSEs.

### Conclusion

Our findings concur with previous reports linking QTLs on chromosomes 2, 4, 6 with TSE incubation time. We also report a novel QTL on chromosome 18. Additionally our results enable us to extrapolate from a previous linkage region on chromosome 11 linked with *ic* transmissions, to a general effect spanning across *ic*/*ip* routes and natural/host adapted prion agents. The generality of this locus may make it a prime candidate for involvement in the general pathogenesis of TSEs, including vCJD. Our demonstration of the substantial selectively with which host genetics may act across different transmission models suggests that not all QTLs identified using the historically favoured *ic* model will feature as strongly for orally acquired TSEs. The enduring aetiological relevance of this route to vCJD contrasts with the apparent decline in the number of cases resulting from blood transfusions [24], [25]. Thus a paradigm shift may be warranted to aid the detection of biological effects specific to vCJD. Such reasoning dictates that *oral* TSE transmissions should be the next priority for mapping studies of this type.

## Materials and Methods

### Ethics statement

Animals were maintained with a 12 hour day/night cycle in a specific pathogen-free (SPF) facility by trained, qualified animal technicians. General husbandry was carried out on a daily basis. Animals had free access to food and water throughout the experiment. The experimental design was vetted and approved by the Home Office (project licence number: PPL 70/4351).

Transmissions were conducted in a category 3 biohazard containment unit using either halothane/oxygen or isoflourane/oxygen for anaesthesia purposes.

Animals were checked daily for neurological symptoms by a research staff member trained and approved in animal handling by the Animal Procedures department of the Home Office. Onset of disease was taken as the onset of three or more concurrent neurological signs. Mice were sacrificed at a defined endpoint of two weeks post diagnosis.

### Mice used in the study

The BXD mouse set is derived from an F2 intercross between C57BL/6 and DBA/2J progenitors. 26 successive generations of brother-sister mating result in the generation of inbred mouselines that are homozygous at virtually every locus [13]. BXD recombinant inbred mouse strains and their progenitors C57BL/6J and DBA2/J were imported from Jackson Laboratories (Bar Harbour, Maine, USA) and maintained by brother-sister mating. All mice were housed under specific pathogen-free conditions for the duration of the experiment. BXDs were routinely tested for homozygosity during the course the study to maintain the integrity of each line. One strain (33ty) was excluded on this basis. F2 mice were generated in-house from an F1 cross. Mice used were uniquely identifiable using subcutaneous transponder tags (AVID, UK).

### Group sizes

Group sizes for the study are given in table 11. Commercial availability of BXD lines determined the number and pattern of strain usage in each experiment. This was mitigated by the survival and proliferation characteristics of each line after importation to UK. BSE *ic* transmissions were heavily prioritised in the F2 study. This was to ensure sufficient numbers to allow meaningful comparisons with previous studies.

**Table 11:** Breakdown of group sizes used.

Experiment	BXD Strains	F2 mice
BSE ic	19	480
BSE ip	18	212
Me7 ic	17	135
Me7 ip	16	123

Group sizes used in BXD and F2 studies.

Adult female mice aged 10–12 weeks old were used. Female mice were used in preference to males as, although group-housing by sex is the only feasible way to maintain a cohort of this size, severe injury and death in males caused by fighting was very frequently observed in a smaller pilot study. The small standard deviations associated with incubation time in BXD mice (see results) suggested a negligible effect of hormonal status.

Age-matched controls were given uninfected brain homogenate at the same dosage and volume as those administered infectious material. Group sizes for controls ranged between 5 and 10. There was no effect of inoculation batch on incubation time for any experiment.

### TSE agents used

BSE affected bovine brain was provided by Central Veterinary Laboratories, Weybridge, UK. Cloned ME7 mouse-adapted scrapie was obtained from BBSRC, UK. Me7 was propagated by passage in C57 animals inoculated *ic*. Brains were harvested at terminal stages of disease.

### Transmissions

#### General procedure

Transmissions were conducted in a category 3 biohazard containment unit. Anaesthesia was carried out using halothane/oxygen. 30 µl of 1% w/v infective brain homogenate was injected into the right parietal lobe for all intra-cerebral (*ic*) inoculations. Intra-peritoneal (*ip*) inoculations used either 100 µl of 10% w/v BSE or 30 µl of 0.1% w/v Me7. Injections were given in the intraperitoneal cavity of the abdominal region. Experimental controls for *ic* and *ip* transmissions were inoculated under the same experimental conditions using identical quantities of uninfected mouse brain material.

#### Dosing strategy

The dosage scheme adopted by this study follows the observation that use of low dose inoculum is associated with increased variability of incubation time [26]. This suggests that it is not feasible to apply the same standard dose to different routes, as this could create bias in the distribution of BXD incubation times. The extent of the bias would be determined by the variable transmission efficiency associated with individual routes. The incubation period data derived from such an approach would actually make traits less suited for evaluations of potential overlap (ie. genetic correlations).

In the worst case scenario, the variability arising from ‘under-dosing’ of routes could mean only a proportion of mice ultimately go on to develop clinical disease within average lifetime. To circumvent this possibility, titration studies were performed during the pilot phase of the study. These were used to establish the minimum dose required to ensure 100% transition from the pre-clinical to clinical disease status within a normal lifespan. These dosages were subsequently adopted for the main study. The advantages of this solution are two-fold:

- constraining of the total phenotypic variance in this way reduces measurement error. This is because data are derived from measurable endpoints, whereas if incubation periods were to exceed natural lifespan of the host it would only be possible to define the clinical endpoint by extrapolation of the observed data.
- the comparability of the resulting incubation period data is increased for genetic correlation purposes, as dosages for each trait are calibrated against a single ‘hit-rate’ target of 100%.

#### Diagnosis

Animals were checked daily for neurological signs. Onset of disease was taken as the appearance of three or more concurrent neurological signs. Symptoms included dysmetria (as assessed by cage-top tests), tremor, wobbly gait and foot clasping [1]. Head tilting, vacant staring and abnormal hyperactivity were other frequent indicators of early end-stage. Mice were sacrificed at a defined endpoint of two weeks post diagnosis. The experiment was terminated sooner for animals whose rate of physical decline warranted this. Incubation time was determined as the number of days between inoculation and establishment of three disease symptoms. Apart from intercurrent illnesss (accountable for death in 7% of cases), all inoculated animals became ill with prion disease.

#### Neuropathology

All neuropathology was performed by the department of Neuropathology at the Institute of Psychiatry, London, UK. Brains of terminally ill animals were removed, fixed in formaldehyde/saline and processed for either haematoxylin and eosin staining or immuno-histochemical staining of PrP deposits using SP40 anti-PrP polyclonal antibody. Confirmation of disease in each of the experimental groups has been achieved by either establishing the existence of PrP-positive amyloid deposits [27] and/or the appearance of neuronal vacuolation in the medulla as in [28]. PrP^sc^ positivity in the medulla was limited to 40% of brain tissues examined. However these animals were recorded as being infected, on the basis of the following:

- Neuronal vacuolation was observed in the medulla of all affected brains.
- No evidence was found of any inflammatory response or other superimposed disease effects.
- Neither injected nor non-injected age-matched controls displayed TSE symptoms (Controls were allowed to go beyond two standard deviations from the tabulated BXD incubation means).
- Scoring was performed blind with respect to affective status (ie. case or control). TSE-related vacuoles were found only in the brains of prion-inoculated mice
- There is no evidence in our data to suggest that the plaque-negative animals form a distinct subgroup in terms of incubation periods (Mann-Whitney U test).

### Statistical analyses

#### General statistical analyses

Basic statistical analyses were carried out using SPSS v13 (LEAD technologies) and MiniTab (MiniTab Inc.).

#### Reliability of BXD data

Internal consistency (or reliability) of the data was estimated in SPSS using the Split-Half method [29]. Estimates were obtained by randomly dividing data for individual strains into two (odds and evens). A split-half coefficient was obtained from the correlation between the two halves. The result (typically over-conservative) is corrected to give a full test score using the Spearman-Brown prophecy formula (SPSS).

#### Genetic calculations

Genetic correlation was investigated in the BXD study by Pearson's correlation of BXD means across traits. This has allowed exploration of genetic overlap between the traits. Calculation of heritability estimated the contribution of genetics overall to each transmission phenotype. Being that members of each BXD line are genetically identical, any variation within each line is ascribed to environmental variance, whereas variance between strains is genetic. One half of the variance of means between the BXD lines estimates additive genetic variance (*V_A_*) while, the mean within-strain variance estimates mean environmental variance (*V_E_*). Thus, an estimate of narrow-sense heritability (h^2^_n_) may be obtained using the formula [30]:
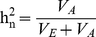
(1)


#### Regression analysis and Interval mapping

QTL analysis was performed by initial linear regression of BXD phenotype data across genotypes at each marker. Simple interval mapping was subsequently implemented using Mapmanager QTX v20b [31] using trait variances to weight BXD strain means.

For F2s interval mapping was performed using consensus map distances from the Mouse Genome database (www.informatics.jax.org). An unconstrained regression model was used with the strength of linkage in each region expressed as a likelihood ratio statistic.

#### Significance thresholds for the genome scan

Genomewide significance thresholds are based on 10,000 permutations of the data (Mapmanager QTX). In order to remove any bias from the computed statistics C57BL/6 and DBA2/J data were excluded from permutation and from simple and composite mapping. Two levels of significance are used to assess the strength of linkage or association in genomewide studies:

The *suggestive* threshold represents the approximate value of the Likelihood Ratio Statistic (LRS) that would be expected to yield on average one false positive per genome scan [17]. In the QTL analysis of BXD mice this corresponds to a genomewide p-value of 0.63 [31]. Though this threshold is very permissive, it is extremely useful for highlighting loci worthy of follow-up. In this case loci reaching suggestive significance will be followed-up in a confirmatory screen that utilises F2 mice. Meanwhile, the *significant* threshold represents the approximate LRS value that would be expected to yield on average one false positive every twenty genome scans [17]. Here this corresponds to a genomewide p-value of 0.05 [31].

#### Composite interval mapping

The WebQTL interface, located at www.genenetwork.org, was used to perform initial interval mapping. This was followed up with a comprehensive analysis of genome-wide linkage using Mapmanager QTXvb20 [31]. Loci meeting nominal significance criteria were interrogated for superimposed effects of other background loci (composite mapping). This was done using multiple regression to establish a panel of background markers under the assumption that constitutively these markers do not act epistatically. A forward step-wise strategy was employed in which the strongest linked markers were added to the background locus model if they improved the fit at the P<0.05 level. QTL Cartographer v2.5 (North Carolina State University) confirmed the location of all F2 QTLs identified in Mapmanager and found a high level of agreement between the observed effects (Spearman's rho  = 0.71, P = 0.0001).

### Genotyping

#### Marker selection

.An advantage of the RI method is that the BXDs are already genetically characterised at over 4000 markers, thus BXD mice require no additional genotyping. For each BXD region nominated for F2 follow-up, markers were chosen on the basis of location and heterozygosity between C57 and DBA progenitors from the Mouse Genome Database (http://www.informatics.jax.org) and the Roche database (http://mousesnp.roche.com). Markers 10 cM either side of each candidate peak were chosen [32]. The phenotypic distribution across the genotypes generally followed the expected 1∶2∶1 ratio with the exception of D6Mit384 this marker was therefore excluded from the BSE *ip* analysis.

#### Polymerase Chain Reaction

DNA was extracted from 1 cm F2 tail samples prior to transmissions using a commercially available kit (Qiagen, cat. No. 69506). 10 ng of DNA (in 5 µl of double-distilled water) was used as the template in 20 µl polymerase chain reactions (PCR). Reactions were carried out in 96-well plates using a PTC-200 thermocycler (GRI, UK). The reaction mix consisted of dNTPs (200 µM), AmpliTaq-Gold and provided buffer (Applied Biosystems), MgCl_2_ (3 mM) and forward/reverse primers (1 picoMolar of each). The 5′ end of each forward primer was fluorescently labelled with either Hex, Fam (MWG Biotech, Germany) or Ned (Applied Biosystems, UK). All reverse primers were obtained from MWG Biotech.

The reaction sequence was as follows: 94°C for 5 minutes; 94°C for 30 seconds, 15 seconds at annealing temperature, 72°C for 30 seconds for 35–40 cycles; 72°C for 5 minutes.

#### Marker Discrimination

PCR products were run in multiplex on a 3100 genetic analyser (Applied Biosystems). Pooled sample were heat denatured in formamide (HiDi, Applied Biosystems) for 2 minutes at 94°C and subsequently snap frozen before being run using a 5 second injection time using a Rox GS-500 size standard (Applied Biosystems). Resolution of individual products within the multiplex required a minimum fluorescence signal of not less than 1000 units per PCR product. The software ‘Genotyper’ (Applied Biosystems) was used to analyse and genotype the output data.

### Meta-analytical methods

#### Pre-processing of data

Implementation of Random-Effects meta-analysis formulae was performed in Microsoft Excel spreadsheets. The effect size r was derived from the estimated variances calculated in Mapmanager, (valid since this equates to r^2^). Conversion of r to Fisher's Z (Zr) is recommended [18], [33]. The conversion is based on the following formula:
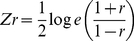
(2)


In practise this is done using a standard conversion table. Differences in the sign associated with a given effect (ie, allelic direction) are accounted for by assigning positive and negative Zr signs for C57BL/6 and DBA/2J alleles respectively.

#### Calculation of heterogeneity

Calculation of heterogeneity was performed using equations appropriate to the number of studies being combined [34]. Equation (3).
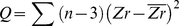
(3)was used for 3 or more studies [18], [34]. Q, the weighted sum of squared errors, has a 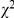
distribution with K-1 degrees of freedom when k equals the number of studies being compared. Equation (4) was used for comparisons of 2 studies (adapted from [34]).
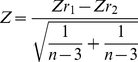
(4)


Equation 4 returns a standard normal deviation (Z) score for which the corresponding p value was obtained using a standard conversion table. This statistic was converted to a 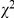 statistic with 1 degree of freedom, equivalent to 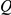 in equation (3).

#### Between-study variance

The between-study variance is an expression of the overall variance between two or more studies, taking into account all of its possible sources. [16] provides equations for estimating the between-study variance (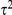) that incorporate the heterogeneity calculated using equations (3) or (4), and a constant (c):
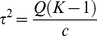
(5)


This was applied in stages so that heterogeneity was first calculated between BXD and F2s for the same trait, and then between traits making up each measure. For example, the measure ‘BSE’ comprises BSE *ic* and BSE *ip*). When BXD and F2 data are initially combined, the constant *c* is calculated using fixed-effect weights as this step occurs before random effects weights are calculated. 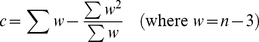
(6)


For subsequent combining between transmissions (for example, Me7 *ic* and Me7 *ip*) random effect weights (w*) are used:
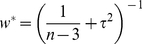
(7)



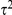
is accumulated over each combination step.

#### The weighted mean effect and its standard error

Both between-study variance (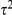) and the within-study variance (n-3) are combined to produce a weighting coefficient (equation 7) for each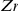. Implementation of the weighting gives the weighted mean effect size, (equation 8) in terms of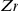. This equation is used to combine this metric across BXD and F2 experiments and between measures:
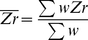
(8)


Actual weighted means are derived after transformation back to r from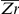. The sampling variance of the average effect size is the reciprocal of the sum of weights [18] therefore the standard error of the average effect size is the square root of the sampling variance, hence:
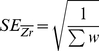
(9)


It is recommended that the Z-score of the weighted mean effect is obtained by dividing the mean effect size by its standard error [18]. This enables the probability associated with r to be calculated via a standard Z to P conversion table.

## Supporting Information

Table S1 Reliability of BXD incubation data per trait. Summary of ‘Split-Half’ reliability estimates. BXD averages are calculated by strain across experiments. Experimental averages are calculated by condition across strains. *These reliability estimates are constrained by low phenotypic variance within the group.
(0.07 MB DOC)

Table S2 Summary of effect sizes derived from Random-Effects meta-analysis procedure. Weighted mean r's for combined BXD and F2s data, r± s.e.m (see methods). Conversion of Zr to r is performed using a standard conversion table. Standard error is calculated as √(1/∑w). *Suggestive (P<8×10^−4^), **Significant (P<2.6×10^-5^).
(0.03 MB DOC)

Table S3 Derived meta-analysis effect sizes summarised by agent/route. Weighted mean r (± s.e.m) for combined BXD and F2s data which is subsequently combined by route and by agent (see methods). *Suggestive (P<8×10^−4^), **Significant (P<2.6×10^−5^).
(0.04 MB DOC)
