# Supplementary material for: Evidence for Varied Aetiologies Regulating the Transmission of Prion Disease: Implications for Understanding the Heritable Basis of Prion Incubation Times
Source: PLoS One. 2010 Dec 2;5(12):e14186. doi: 10.1371/journal.pone.0014186 (PMC2996284; doi:10.1371/journal.pone.0014186)
Supplement: Table S1 — Reliability of BXD incubation data per trait. Summary of ‘Split-Half’ reliability estimates. BXD averages are calculated by strain across experiments. Experimental averages are calculated by condition across strains. *These reliability estimates are constrained by low phenotypic variance within the group. (0.07 MB DOC) [file pone.0014186.s001.doc]

| **Strain** | **BSE ic** | **BSE ip** | **Me7 ic** | **Me7 ip** | **BXD averages** |
| --- | --- | --- | --- | --- | --- |
|
| BXD1 | 0.98 | 0.93 | - | - | 0.96 |
| BXD2 | 0.97 | - | - | 0.93 | 0.95 |
| BXD6 | 0.97 | 0.86 | 0.79 | 0.95 | 0.90 |
| BXD8 | 0.81 | 0.99 | 0.83 | 0.91 | 0.89 |
| BXD9 | 0.92 | 0.98 | 0.91 | 0.96 | 0.94 |
| BXD11 | 0.91 | 0.86 | 0.99 | 0.99 | 0.94 |
| BXD12 | 0.91 | 0.97 | 0.93 | 0.89 | 0.93 |
| BXD14 | 0.99 | 0.99 | 0.9 | 0.9 | 0.93 |
| BXD19 | 0.87 | 0.91 | 0.92 | 0.99 | 0.93 |
| BXD21 | 0.79 | 0.99 | 0.99 | 0.99 | 0.94 |
| BXD22 | 0.96 | 0.33* | 0.92 | 0.87 | 0.74 |
| BXD23 | 0.99 | 0.93 | 0.96 | 0.94 | 0.95 |
| BXD24 | 0.98 | 0.96 | 0.86 | 0.93 | 0.93 |
| BXD25 | 0.99 | 0.72 | 0.97 | 0.97 | 0.91 |
| BXD27 | 0.84 | 0.96 | 0.58* | 0.94 | 0.72 |
| BXD28 | 0.61 | 0.99 | 0.99 | - | 0.87 |
| BXD31 | 0.89 | 0.86 | 0.96 | 0.98 | 0.92 |
| BXD32 | 0.95 | 0.99 | 0.74 | 0.78 | 0.86 |
| C57 | 0.98 | 0.89 | 0.81 | 0.98 | 0.92 |
| DBA | 0.91 | 0.91 | 0.97 | 0.99 | 0.94 |
| **Exptl Average** | 0.91 | 0.88 | 0.86 | 0.94 |  |
