# Supplementary material for: Evidence for Varied Aetiologies Regulating the Transmission of Prion Disease: Implications for Understanding the Heritable Basis of Prion Incubation Times
Source: PLoS One. 2010 Dec 2;5(12):e14186. doi: 10.1371/journal.pone.0014186 (PMC2996284; doi:10.1371/journal.pone.0014186)
Supplement: Table S2 — Summary of effect sizes derived from Random-Effects meta-analysis procedure. Weighted mean r's for combined BXD and F2s data, r± s.e.m (see methods). Conversion of Zr to r is performed using a standard conversion table. Standard error is calculated as √(1/∑w). *Suggestive (P<8×10^−4^), **Significant (P<2.6×10^-5^). (0.03 MB DOC) [file pone.0014186.s002.doc]

|  |
| --- |
| | Experiment | QTL LOCATION | | | | | | | | | | | --- | --- | --- | --- | --- | --- | --- | --- | --- | --- | --- | | Chr 1 | Chr 2 | Chr 3 | Chr 4 | Chr 6 | Chr 8 | Chr 10 | Chr 11 | Chr 14 | Chr 18 | | Me7ip | -0.33 ± 0.11 | -0.24 ± 0.12 | 0.15 ± 0.06 | 0.22 ± 0.12 | 0.19 ± 0.10 | 0.08 ± 0.09 | -0.21 ± 0.08 | -0.14 ± 0.06 | 0.15 ± 0.06 | 0.25 ± 0.14 | | ME7ic | 0.20 ± 0.06***** | -0.22 ± 0.06***** | -0.10 ± 0.10 | -0.30 ±0.06****** | 0.10 ± 0.10 | 0.17 ± 0.60 | -0.05 ± 0.10 | -0.19 ± 0.06***** | 0.15 ± 0.09 | 0.04 ± 0.09 | | BSEip | 0.29 ± 0.07***** | -0.19 ± 0.10 | -0.14 ± 0.07 | -0.09 ± 0.08 | 0.00 ± 0.05 | 0.11 ± 0.07 | 0.20 ± 0.08 | -0.23 ± 0.05****** | 0.11 ± 0.07 | -0.22 ± 0.05****** | | BSE ic | 0.16 ± 0.05 | -0.11 ± 0.06 | -0.08 ±0.05 | -0.19 ± 0.05***** | 0.15 ± 0.04***** | 0.05 ± 0.05 | 0.10 ± 0.05 | -0.15 ± 0.04***** | 0.03 ± 0.05 | 0.07 ± 0.06 | |
