# Supplementary material for: Evidence for Varied Aetiologies Regulating the Transmission of Prion Disease: Implications for Understanding the Heritable Basis of Prion Incubation Times
Source: PLoS One. 2010 Dec 2;5(12):e14186. doi: 10.1371/journal.pone.0014186 (PMC2996284; doi:10.1371/journal.pone.0014186)
Supplement: Table S3 — Derived meta-analysis effect sizes summarised by agent/route. Weighted mean r (± s.e.m) for combined BXD and F2s data which is subsequently combined by route and by agent (see methods). *Suggestive (P<8×10^−4^), **Significant (P<2.6×10^−5^). (0.04 MB DOC) [file pone.0014186.s003.doc]

| Experiment | QTL LOCATION | | | | | | | | | |
| --- | --- | --- | --- | --- | --- | --- | --- | --- | --- | --- |
|  | Chr 1 | Chr 2 | Chr 3 | Chr 4 | Chr 6 | Chr 8 | Chr 10 | Chr 11 | Chr 14 | Chr 18 |
| ic Expts | 0.17 ± 0.25 | -0.16 ± 0.17 | -0.09 ± 0.23 | -0.23 ± 0.26 | 0.14 ± 0.22 | 0.12 ± 0.14 | 0.02 ± 0.22 | -0.18 ± 0.03****** | 0.10 ± 0.20 | 0.06 ± 0.23 |
| ip Expts | 0.002 ± 0.70 | -0.22 ± 0.35 | 0.01 ± 0.32 | 0.04 ± 0.51 | 0.05 ± 0.49 | 0.09 ± 0.24 | -0.01 ± 0.39 | -0.18 ± 0.06 | 0.13 ± 0.24 | -0.03 ± 0.62 |
| Me7 Expts | 0.06 ± 0.36 | -0.22 ± 0.20 | 0.03 ± 0.24 | -0.08 ± 0.33 | 0.14 ± 0.33 | 0.14 ± 0.15 | -0.11 ± 0.25 | -0.18 ± 0.04****** | 0.15 ± 0.18 | 0.11 ± 0.36 |
| BSE Expts | 0.22 ± 0.28 | -0.15 ± 0.21 | -0.11 ± 0.20 | -0.14 ± 0.25 | 0.08 ± 0.08 | 0.08 ± 0.17 | 0.14 ± 0.22 | -0.19 ± 0.05* | 0.07 ± 0.20 | -0.07 ± 0.20 |
| **All Expts** | **0.08 ± 0.27** | **-0.19 ± 0.21** | **-0.04 ± 0.21** | **-0.09 ± 0.24** | **0.11 ± 0.27** | **0.10 ± 0.16** | **0.01 ± 0.22** | **-0.18 ± 0.03**** | **0.11 ± 0.19** | **-0.04 ± 0.29** |
